# Buckling-Based Non-Linear Mechanical Sensor

**DOI:** 10.3390/s18082637

**Published:** 2018-08-11

**Authors:** Sangmin An, Bongsu Kim, Soyoung Kwon, Geol Moon, Manhee Lee, Wonho Jhe

**Affiliations:** 1 Department of Physics & Astronomy, Seoul National University, Seoul 08826, Korea; rolliney@hanmail.net (B.K.); duddl333@snu.ac.kr (S.K.); 2 Department of Physics, Chonnam National University, Gwangju, Chonnam 61186, Korea; cnuapi@jnu.ac.kr; 3 Department of Physics, Chungbuk National University, Cheongju, Chungbuk 28644, Korea; mlee@cbnu.ac.kr

**Keywords:** buckling cantilever, non-linear force sensor, atomic force microscopy, bifurcation

## Abstract

Mechanical sensors provide core keys for high-end research in quantitative understanding of fundamental phenomena and practical applications such as the force or pressure sensor, accelerometer and gyroscope. In particular, in situ sensitive and reliable detection is essential for measurements of the mechanical vibration and displacement forces in inertial sensors or seismometers. However, enhancing sensitivity, reducing response time and equipping sensors with a measurement capability of bidirectional mechanical perturbations remains challenging. Here, we demonstrate the buckling cantilever-based non-linear dynamic mechanical sensor which addresses intrinsic limitations associated with high sensitivity, reliability and durability. The cantilever is attached on to a high-*Q* tuning fork and initially buckled by being pressed against a solid surface while a flexural stress is applied. Then, buckling instability occurs near the bifurcation region due to lateral movement, which allows high-sensitive detection of the lateral and perpendicular surface acoustic waves with bandwidth-limited temporal response of less than 1 ms.

## 1. Introduction

Since the first introduction by Leonhard Euler [1], the phenomenon of buckling, a mechanical instability leading to flexural fracture under high compressive stress, has been widely studied in regard to, for example, the compressibility of living cells [2], the bending and buckling of carbon nanotubes [3], and the buckling of thin films on mismatched substrates [4] and various materials [5,6,7]. In particular, flexural rigidity and elastic instability cause non-linear responses and complex behaviors which have been investigated for thermal expansion [8], elastohydrodynamic instabilities [9], coil-stretch transition [10] and buckling of elastic filaments [11]. Interestingly, recent studies have shown that buckling instability produces greatly enhanced sensitivity of external mechanical disturbances and has been recently studied for understanding related effects, including buckling of hair-cell tip links in animals’ ears with elastic channel transmission of the force [12] and the biomimetic force sensor mimicking an auditory hair cell [13].

A mechanical sensor converts from physical disturbances such as force, pressure, tactile [14], velocity [15,16], inertia [17], flow [18], acceleration and inertial vibration, to observable signals. These converting techniques allow great improvements to sensitive measurement [19,20,21,22,23]. A seismometer employing a zero-length spring [24] or force balance scheme [25] is representative of requiring a highly sensitive detection for the early warning of earthquakes to protect from destructive shaking [26]. However, their sensitivity is limited by intrinsic mechanical properties such as the spring constant, resonance frequency, quality (*Q*-) factor of relatively massive harmonic oscillators (mass of several kg) and unsuitability of the stable steady state as linear responsible devices. Here, we introduce the atomic force microscope-based non-linear mechanical sensor using a combination system of the buckling cantilever and quartz tuning fork-based atomic force microscope (QTF-AFM). This allows fast, accurate, sensitive and quantitative dynamic force measurement due to bifurcation-enhanced sensing technique which results in high sensitivity with a relatively fast response time. In addition, this system provides simultaneous sensing of both perpendicularly (P-) and laterally (L-) transferred perturbations.

## 2. Materials and Methods

**Figure 1:**
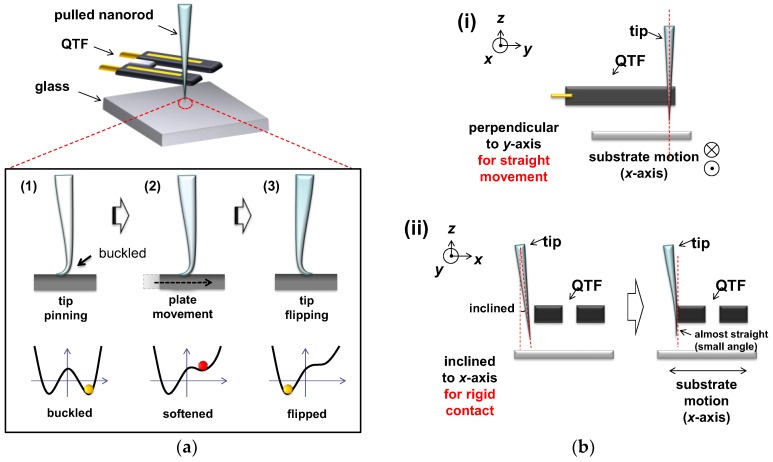
Bucking cantilever-based mechanical sensor: (**a**) experimental procedure is as follows: (1) the sharp cantilever (pulled quartz nanorod) attached on the one prong of the quartz tuning fork (QTF) with apex diameter of ~150 nm, approaches the substrate until contact, gently pushes further to become buckled under flexural stress. (2) The plate moves laterally and the tip experiences softening with a noise increment at the bifurcation region, which states make a non-linear sensitive mechanical sensor. (3) Finally, the abrupt flipping of the tip triggered by its lateral movement or an external impact of mechanical disturbance; (**b**) the tip installed perpendicular to *y*-axis for straight movement and inclined to *x*-axis for rigid contact.

The buckling cantilever-based non-linear mechanical sensor, which employs a cantilever (pulled quartz nanorod) attached to the QTF-AFM, detects external disturbances (Figure 1a). The experimental procedure is as follows: (1) approach of the cantilever and contact on the clean glass (Pyrex glass, 1.5 cm × 1.5 cm, 200 μm thickness) substrate; (2) further pushing until buckling; and (3) abrupt flipping by a slight lateral movement or a small impact. The tip is in the elastic regime, as evidenced by the fact that it straightens up after the stress is released. Note that, at the procedure of (1), the direction where buckling occurs is determined by the instantaneous direction of the contact force acting on the substrate, which is almost perpendicular to the surface but with an error of 2~3% in tilt angle. Namely, the tip leans against the fork which breaks the symmetry by applying unidirectional stress. This leads to buckling in the direction of the stress so that one side is stretched and the other is squeezed by the QTF. In detail, we used a highly magnified charge coupled device (CCD) camera to make a precise tip–QTF alignment with respect to the substrate in the *x*, *y*, and *z*-axes. We first installed the tip onto an *x*-*y*-*z* translator for its accurate attachment to a QTF prong in the direction perpendicular to the *y*-axis (i.e., normal to the substrate) for straight movement of the substrate in the *x* direction (Figure 1(bi)) and for its hard contact to one prong of the QTF with its initial small contact angle in the *x*-axis (Figure 1(bii)). The tip that is initially slightly inclined in the *x*-axis becomes almost straight and normal to the substrate by a manual hard push toward the prong, which nonetheless has a small alignment error of a few degrees. This range of angular error affects the flipping point by lateral movement within an error range of under 3%, with sensitivity variation under 5%, reliably and repeatedly. Normally a conventional AFM cantilever is installed perpendicular to the substrate, but this system has an angular error for the purpose of avoiding tip wear or shaving with preventing direct tip contact to the surface. The exerted push force associated with such a hard contact is performed while the QTF stiffness is about 27,000 N/m, and thus rigid contact of the tip on the QTF is secured without any change of the tip–QTF contact area due to the interaction force between the tip apex and the substrate. Note that the local buckling on the tip is elastic, thus a nearly vertical approach led to soft contact and subsequent buckling of the tip on the substrate without plastic deformation of the tip apex, which was confirmed by the optical microscope (OM) images showing the tip having recovered its straightness after retraction from the buckled condition. The cantilever with an apex diameter of ~150 nm is fabricated by pulling a 1 mm diameter quartz rod with a mechanical puller (P-2000, Sutter Instruments, Novato, CA, USA), and attached to one prong of the QTF sensor, which provides detailed information on the interactions. The fabrication process of the pulled pencil-shaped nanorod is as follows: (i) holding both sides edge of 1 mm diameter quartz rod; (ii) focusing a high-energy CO_2_ laser in the middle of the quartz rod for melting; (iii) pulling both sides to fabricate the pencil-shaped sharp cantilever with an apex diameter of nanometer scale. Normally, one can choose the material of a 1 mm rod as quartz or borosilicate rod for a commercial puller. We chose the quartz rod, which has a relatively high Young’s modulus for reliable and repeatable usage. When we magnified the tip apex region, the tapered angle of the pencil was about 2°, therefore the tip easily buckled by surface contact and further pushing. We expect that the variations in tapered angles or materials influence the sensitivity of the system, so that the optimal tapered angle could maximize the experimental sensitivity.

## 3. Results

The mechanical response of the buckling cantilever was experimentally obtained by a frequency modulation (FM)-mode QTF-AFM system [27]. We can obtain the associated elastic (*F*_k_) and viscous forces (*F*_b_) and energy dissipation (*E*_dis_) by using the elasticity (*k*_int_) and damping coefficient (*b*_int_), calculated from the measured frequency shift (Δ*F*) and damping coefficient (*g*’) within the theory of FM QTF-AFM. Briefly, we obtained *F*_k_ and *F*_b_ from the measured Δ*F* and *g*’ as $F_{k}=k_{\mathrm{int}}A_{0}$ and $F_{b}=b_{\mathrm{int}}\omega_{0}A_{0}$ with $k_{\mathrm{int}}=k\left[{\left(1+\frac{\Delta F}{f_{0}}\right)}^{2}-1\right]$, $b_{\mathrm{int}}=k/Q\omega_{0}\left(\frac{g\prime }{g}\frac{f_{0}}{f_{0}+\Delta F}-1\right)$, where *A*_0_ is the oscillation amplitude of the QTF, ω_0_ (=2π*f*_0_); *f*_0_ is eigen-frequency, *k* is the QTF stiffness (~27,000 N/m), *Q* is the quality factor, and g is initial damping.

### 3.1. Sensitivity Increment at the Region of Bifurcation

**Figure 2:**
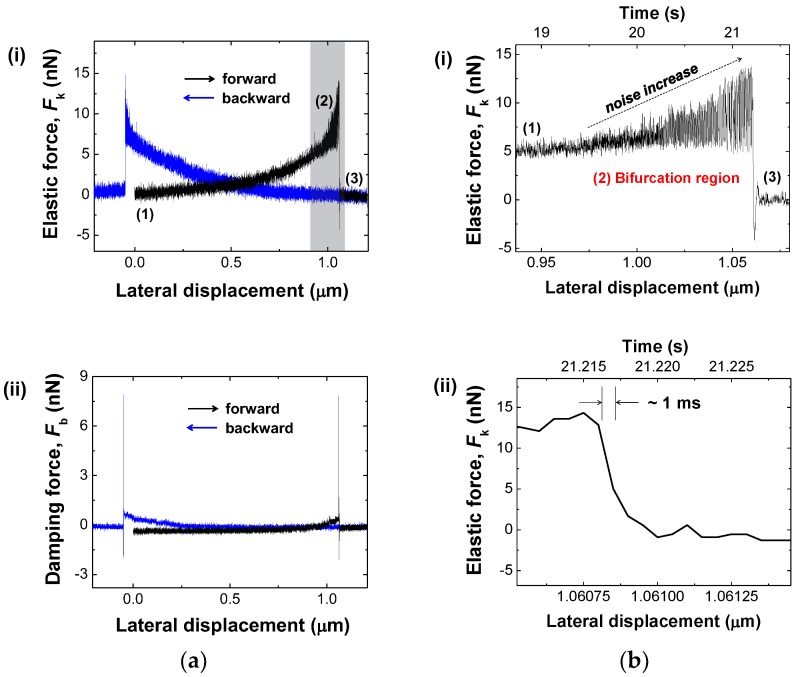
Instability-induced flipping of buckling cantilever by lateral movement: (**a**) during lateral movement of 1 μm distance (for duration of ~22 s at the tip speed of 50 nm/s), the tip experiences accumulated flexural forces and the resulting enhancement of non-linear fluctuations triggers random switching of the buckling direction with decrement of potential wall. The continuous accumulation of the forces results in the corresponding increment of the Elastic force (*F*_k_-(i)) and damping force (*F*_b_-(ii)) in the frequency modulation (FM) detection of a QTF-based atomic force microscope (AFM); (**b**) magnified gray area of (**a**). (i) The tip with a given initial buckling (1) experiences increment of forces until it reaches the bifurcation region by lateral movement (2), and within the region of enhanced non-linear fluctuations, the tip flips abruptly accompanying recovery of the initial frequency but with the buckling direction reversed (3). (ii) Fast time-resolved mechanical responses resulting from abrupt flipping (~1 ms in 0.2 nm distance) are observed.

Figure 2a shows the experimental response of the buckling cantilever during its lateral movement: elastic force (*F*_k_-(ii)) and damping force (*F*_b_-(ii)) which are derived by calculating from the raw data of the frequency shift (Δ*F*) and the damping coefficient (*g*’). *F*_k_ and *F*_b_ slightly but gradually increase due to an increment of the responding force on the surface by lateral movement of the buckling cantilever, until the tip flips abruptly. Figure 2(bi) is the magnified gray area of Figure 2(ai). We clearly observe the increased fluctuation of *F*_k_ in the region of bifurcation right before the flipping point, which is attributed to the enhanced mechanical instability of the buckling cantilever [28,29]. Note that we derive *Q*-factor variation from Δ*F* and *g*’ changes when the tip moves laterally (*Q*/*Q*’ = (1 + Δ*F/f*_0_) *g/g*’). Experimentally obtained Δ*F* is much less than resonance frequency (*f*_0_), and thus *Q*-factor decreases due to the increment of *g*’. Therefore, the increment of instability is not from increment of the *Q*-factor but decrement of activation energy barrier near the transient state which induces high sensitivity. When the initially buckling cantilever reaches the flipping, the potential energy barrier gradually decreases. Thus, extremely small perturbations in energy cause tip flipping. Namely, in the region of bifurcation, interacted forces dramatically change the response signal due to the fluctuations of amplitude and phase responses of the oscillator, which results in instability of the system with high sensitivity. Notice that while the buckling cantilever is a spatial symmetry-broken state, the symmetry is restored at the bifurcation region, and thus the tip has an equal probability to transit to the right or left side. Another important observation is that the measured response time associated with the mechanical flipping is found as fast as 1 ms (Figure 2(bii)), which is just the upper limit of the FM QTF-AFM detection system [30], indicating the flipping time can be shorter than 1 ms.

### 3.2. Characterization of the Non-Linear Sensor

**Figure 3:**
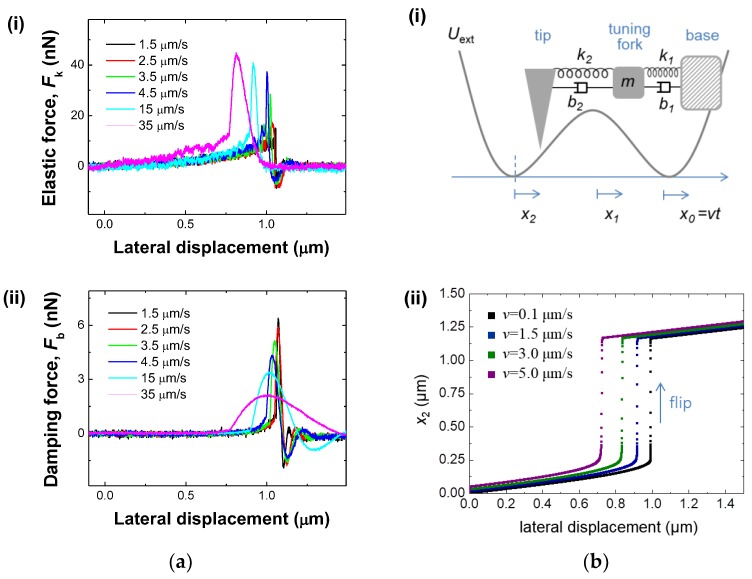
Speed dependence of mechanical responses by the buckling cantilever: (**a**) the mechanical sensor detects the elastic forces (*F*_k_-(i)) as well as the viscous forces (*F*_b_-(ii)) while the buckling cantilever experiences abrupt flipping during lateral movement of the tip with the tip speed of 1.5 μm/s~35 μm/s. The buckling cantilever starts to sense the interaction away from the flipping point as the speed of the tip movement increases; (**b**) non-linear transition model. (i) The coupled cantilever–oscillator model, where the tip is present in double-well potential *U*_ext_ and connected with the tuning fork and the tuning fork base moves at a constant velocity *v*. (ii) Non-linear transient dynamics of the tip for different normalized velocities of the base: *v* = 0.1, 1.5, 3, and 5 μm/s. With increasing *v*, the flipping point decreases at which the static bifurcation occurs, which is consistent with experimental results.

For further characterization of the buckled system, we have investigated the speed dependence of the buckling cantilever-based mechanical sensor. Figure 3a plots the elastic (*F*_k_)-(i) and viscous (*F*_b_)-(ii) forces versus the lateral movement for the tip speed from 1.5 μm/s to 35 μm/s. Seeing the behavior of *F*_k_ (Figure 3(ai)) as a function of the distance variation, the buckling cantilever starts to sense the interaction away from the flipping point as the speed of the tip movement increases, which indicates the response of the system is sensitive enough when tip stays within the bifurcation region. Note that in the case of slow movement (<2.5 μm/s), the tip stays longer at the bifurcation region with increment of noise storage which results in flipping like noise-induced flipping. The apex of the tip whose stiffness is about 20 N/m experiences tip softening along with the lowering of the energy barrier for fast tip movement (>3.5 μm/s). Note that the elastic force becomes stronger at the higher tip speed after 3.5 μm/s, which indicates that the repulsive elastic energy is slowly increasing away from the bifurcation region but rapidly increasing near the bifurcation region due to the higher tip speed. In the case of viscous force (Figure 3(aii)), it gets weaker at the higher tip speed due to the less damped interaction with the surface for fast movement and relaxes faster with the increment of tip speed after the flipping occurs, which indicates that while the maximum viscous energy is more rapidly damped away as the buckling cantilever approaches the bifurcation region. This phenomenon may be associated with the energy-damping rate. Normally, with a conventional AFM there is an optimal scanning speed for ideal scan images. In view of this, we can think about an optimal speed for ideal sensitivity, the magnitude of elastic or viscous responses, and the flipping point of the system. For sensitivity in the experiment, we defined the sensitivity on a stop position on the region of bifurcation for sensing the perturbations, thus speed-dependent sensitivity variation is out of the scope of the work. In addition, within the performed speed under 35 μm/s, the elastic and viscous tip responses of the tip show increasing and decreasing behaviour with decrement of the flipping point.

To understand the non-linear transient dynamics of the tip, we model the system as a compliant tip in a double-well potential *U*_ext_, where the tip is coupled to the tuning fork and the base of the tuning fork is slowly drawn at a constant velocity *v*, as described in Figure 3(bi). We assume the mass of the tip is much smaller than that of the tuning fork and is set to zero for simplicity, as similarly assumed in the AFM study of stick-slip motion [31]. Two minima of the double-well potential *U*_ext_ correspond to two buckled states of the tip. Total potential energy of the system is given as:

(1) $$U_{\mathrm{tot}}=\frac{1}{2}k_{1}{\left(x_{1}-x_{0}\right)}^{2}+\frac{1}{2}k_{2}{\left(x_{2}-x_{1}\right)}^{2}+U_{\mathrm{ext}}\left(x_{2}\right)$$

where *k*_1_ and *k*_2_ are the stiffness constants of the tip and the tuning fork, and *x*_1_ and *x*_2_ are the positions of the tip and the tuning fork, respectively. Since the tuning-fork stiffness (~25,000 N/m) is much greater than that of the tip (~20 N/m), we can approximate *x*_1_ ≈ *x*_0_ during the base movement, so that total potential energy becomes $U_{\mathrm{tot}}\approx 1/2k_{2}{\left(x_{2}-x_{1}\right)}^{2}+U_{\mathrm{ext}}\left(x_{2}\right)$. Note that the position of the base moves at a constant velocity such as *x*_0_ = *vt*, and thus *U*_tot_ changes accordingly. To study the velocity dependence of the flip transition, we derive the equation of motion for the tip from the Lagrange equation with the potential energy *U*_tot_ as:

(2) $$b_{2}{\dot{x}}_{2}+k_{2}x_{2}=F_{\mathrm{ext}}\left(x_{2}\right)+k_{2}vt+b_{2}v$$

where $F_{\mathrm{ext}}=-dU_{\mathrm{ext}}/dx$. Figure 3(bii) shows the numerical solutions of Equation (2) for various velocities *v* = 0.1, 1.5, 3, and 5 μm/s. Increasing the base velocity *v*, we find the distance that the system travels before the flip decreases (blue, green, and purple dots). In other words, when the excitation parameter, *v* in the present case, is not stationary, the transition occurs away from the point where the static transition occurs, as consistently observed in the experiments (Figure 3(ai)). These results indicate the sensor has the unique characteristics of a non-linear oscillator that allows high sensitivity.

### 3.3. Demonstration of Senstive Mechnical Sensor

**Figure 4:**
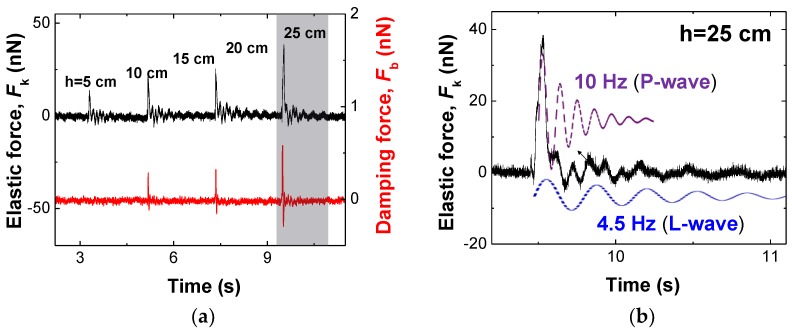
Detection of polarization-dependent sensitive mechanical disturbances: (**a**) after the buckling cantilever placed on the region of bifurcation for continuous detection, we sense continuously the signals of *F*_k_ and *F*_b_ generated by the disturbances from the nearby impact spot where a coin (mass of ~5.5 g) is dropped at four different release heights (10 cm and 25 cm), corresponding to the higher impact strength for the latter case; (**b**) magnified graph of 25 cm case. Two orthogonally polarized mechanical waves are simultaneously detected at different oscillation frequencies, P-wave (10 Hz) and L-wave (4.5 Hz), propagating through the massive stage system on which the buckling-tip sensor is tightly fixed, as shown in the detailed temporal responses given in each lower panel.

We demonstrate the buckling cantilever for sensitive, quantitative and simultaneous detection of the polarization-dependent P-wave and L-wave in Figure 4. We placed the tip in the middle of bifurcation region (noise increased area). The buckling cantilever is ready to measure the interaction forces via continuous detection of the mechanical waves transferred from the impact position on the system stage, whereby we drop a coin (mass of ~5.5 g) while increasing the release height from 5 cm to 25 cm (Figure 4a). We observe *F*_k_ increase with increment of *F*_b_ with respect to the dropping height. For the case of 25 cm height (Figure 4b), the gravitational potential energy is 1.348 × 10^−2^ N·m, which is transferred through the mass of the entire detector stage floating in air on a small vibration isolator. Most of the coin’s kinetic energy is absorbed by the system, while the remaining energy exerts forces onto the buckling cantilever, which are detected as the elastic and viscous forces. We find the values of *F*_k_ and *F*_b_ in the 25 cm case are about 3 times larger than the 10 cm case.

In a closer look at the measured *F*_k_ and *F*_b_ in both cases, we observe two different damped oscillatory mechanical waves of ~10 Hz (P-wave) and ~4.5 Hz (L-wave), respectively. The measured frequency of P-(~10 Hz) and L-(~4.5 Hz) waves came from perpendicularly and laterally oscillation caused by the small-sized air-floating anti-vibration chamber table’s resonance, where the measurement AFM system is facilitated. This is a reason why we sense their frequencies. For further investigation of varying perturbed frequency with dynamic range, we need to test in the real environment of an earthquake, which is beyond the proposed work. We expect the sensor can detect the earthquake’s perturbation frequencies (20~20,000 Hz) with the nanorod-combined QTF sensor’s resonance frequency which is about 32 kHz. Notice that different frequency wave typically produces different wave-transferring velocity in an earthquake such as different speeds of P and S waves. However, in the experiment, because the distance between the impact spot of coin and the buckling cantilever is short, the time intervals of the P-waves and L-waves are relatively short and thus we could observe their superimposed responses. Note that one can reach the minimum detection force limit of ~10^−14^ N ($dF/dz_{min}=\sqrt{4k_{eff}k_{B}TB/\left(Q\omega_{0}A_{0}^{2}\right)}$, where $k_{eff}$ is effective tip stiffness near the apex of the pulled rod, B is bandwidth, Q is *Q*-factor, $A_{0}$ is amplitude of the tip oscillation) parking the buckled tip on the bifurcation region with compensating for the noise issue. Thus, with extremely small perturbation above this potential energy, the buckling cantilever can be flipped with detection capability of P-(~10 Hz) and L-(~4.5 Hz) waves whose response is critically suppressed by flipping to the stable state of the tip. The sensitivity is a critical factor for general mechanical sensors, which is typically compared via the gauge factor, the ratio of change in electrical resistance to mechanical strain. In particular, seismometers are described in the unit of V/m/s and typical value is about 2000 V/m/s [32]. In our case, however, we could obtain over ~10^6^ V/m/s for continuous sensing near the bifurcation region, which is derived from the output voltage of the sensor and the wave velocity (i.e., displacement per duration of the stage motion). This demonstrates sensitive detection of extremely small external perturbations, even the faint precursor waves before a strong impact such an earthquake.

### 3.4. Reliability and Durability

**Figure 5:**
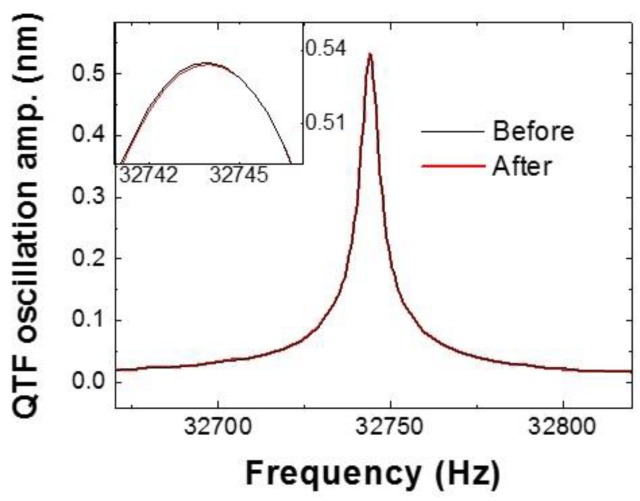
Reliability and durability of the buckling cantilever: We measured the resonance curves of the sensor before and after 100 oscillations. Both resonance frequencies are almost identical, at the center frequency of 32,748 Hz with a shift of ~10^−4^ Hz, and both *Q*-factors are 10,520, which indicates tip wear of ~10^−4^ % with the boundary of the diameter of the tip apex, thus 10^7^ oscillation gives no noticeable wear.

For verification of the sensor, we measured the resonance curves before (i.e., while unbuckled in air) and after (i.e., in buckled condition) 100 continuous flips in order to check the reliability and durability of the tip at the moving speed of 15 µm (Figure 5). We found that resonance frequencies are virtually identical, having a center frequency of 32,748 Hz within a shift of ~10^−4^ Hz, and also both *Q*-factors are 10,520 (±2.3) before and after 100 flips; 100 times oscillation results in the mass change of ~10^−13^ g and tip wear of only ~0.4%, which indicates that the system can make ~10^4^ flips at 15 µm/s without any noticeable wear of the tip. Even if distinct wear exists, we could sense the internal interaction clearly. Note that the sensor has a capability of accurate mass measurement with a resolution better than ~10^−13^ g, as derived from the measurable error of the effective stiffness (*dk*_int_) and angular frequency (ω^2^); *dk*_int_/ω^2^. We assume *dk* ~ 0.1 N/m in ambient conditions gives a detectable mass resolution of ~10^−13^ g.

## 4. Conclusions

In conclusion, we have demonstrated a buckling cantilever-based non-linear mechanical sensor which allows highly sensitive and polarization (or direction) dependent fast detection. For practical application, for example, as an earthquake sensor, this system has to demonstrate primary and secondary sensing capability, as well as love and Rayleigh waves, which may be feasible, compared to other similar schemes that use QTFs. In addition, we can control the tip’s position near the bifurcation region automatically for detecting repeatedly weak mechanical disturbances with decent data analysis based on durable, reliable and continuous (or repeatable) detection.
